# Time- and dose-dependent high-sensitivity cardiac troponin-T to improve outcome prediction after TAVI: a multicenter cohort study

**DOI:** 10.1007/s00392-025-02808-z

**Published:** 2025-11-24

**Authors:** Thorald Stolte, Jakob Johannes Reichl, Pedro Lopez-Ayala, Ivo Strebel, Felix Goetzinger, Max Wagener, Jasper Boeddinghaus, Gregor Leibundgut, Ramona Schmitt, Dirk Westermann, Tau Hartikainen, Christian Mueller, Felix Mahfoud, Philipp Ruile, Philipp Breitbart, Thomas Nestelberger

**Affiliations:** 1 Department of Cardiology and Cardiovascular Research Institute Basel (CRIB), University Hospital Basel, University of Basel, Basel, Switzerland; 2 Department of Cardiology and Angiology, Medical Center, Faculty of Medicine, University of Freiburg, University of Freiburg, Südring 15, 79189 Bad Krozingen, Germany

**Keywords:** Aortic stenosis, Transcatheter aortic valve implantation, High-sensitivity cardiac troponin T, Periprocedural myocardial injury

## Abstract

**Background:**

Pre- and post-procedural high-sensitivity cardiac troponin T (hs-cTnT) detects periprocedural myocardial injury (PPMI) and predicts adverse outcomes following transcatheter aortic valve implantation (TAVI). Current diagnosis of PPMI relies on fixed cutoffs, lacking the integration of time- and dose-dependent effects of hs-cTnT. This limits the precision of risk stratification and subsequent patient management.

**Aims:**

To investigate the non-linear and time-dependent effects of pre- and post-procedural hs-cTnT levels on outcomes after TAVI, these findings were compared to the dichotomized definition of PPMI proposed by the Valve Academic Research Consortium-3 (VARC-3).

**Methods:**

Consecutive patients undergoing TAVI between 2011 and 2024 at two tertiary university hospitals with available hs-cTnT measurements were enrolled. The primary outcome was all-cause mortality at 1 year. Multivariable Cox proportional hazards models were fitted. To relax the proportional hazards assumption, allowing for hazard ratios (HRs) to vary over time and across hs-cTnT values, a Royston–Parmar model was fitted.

**Results:**

Among 5158 patients, the HR for all-cause mortality at 1 year associated with VARC-3 defined PPMI was not statistically significant. Continuous variable analysis showed that both higher pre- and post-procedural hs-cTnT levels correlated with increased all-cause mortality risk at 1 year. Time-dependent models revealed the hazard to be greatest for higher hs-cTnT levels early post-procedurally and to decline over time.

**Conclusions:**

The dichotomized VARC-3 definition of PPMI showed no prognostic value. Modelling hs-cTnT as continuous and time-dependent revealed a dynamic risk trajectory after TAVI. Incorporating these non-linear and time-dependent effects into risk prediction models may improve clinical decision-making and personalize post-procedural surveillance.

**Graphical Abstract:**

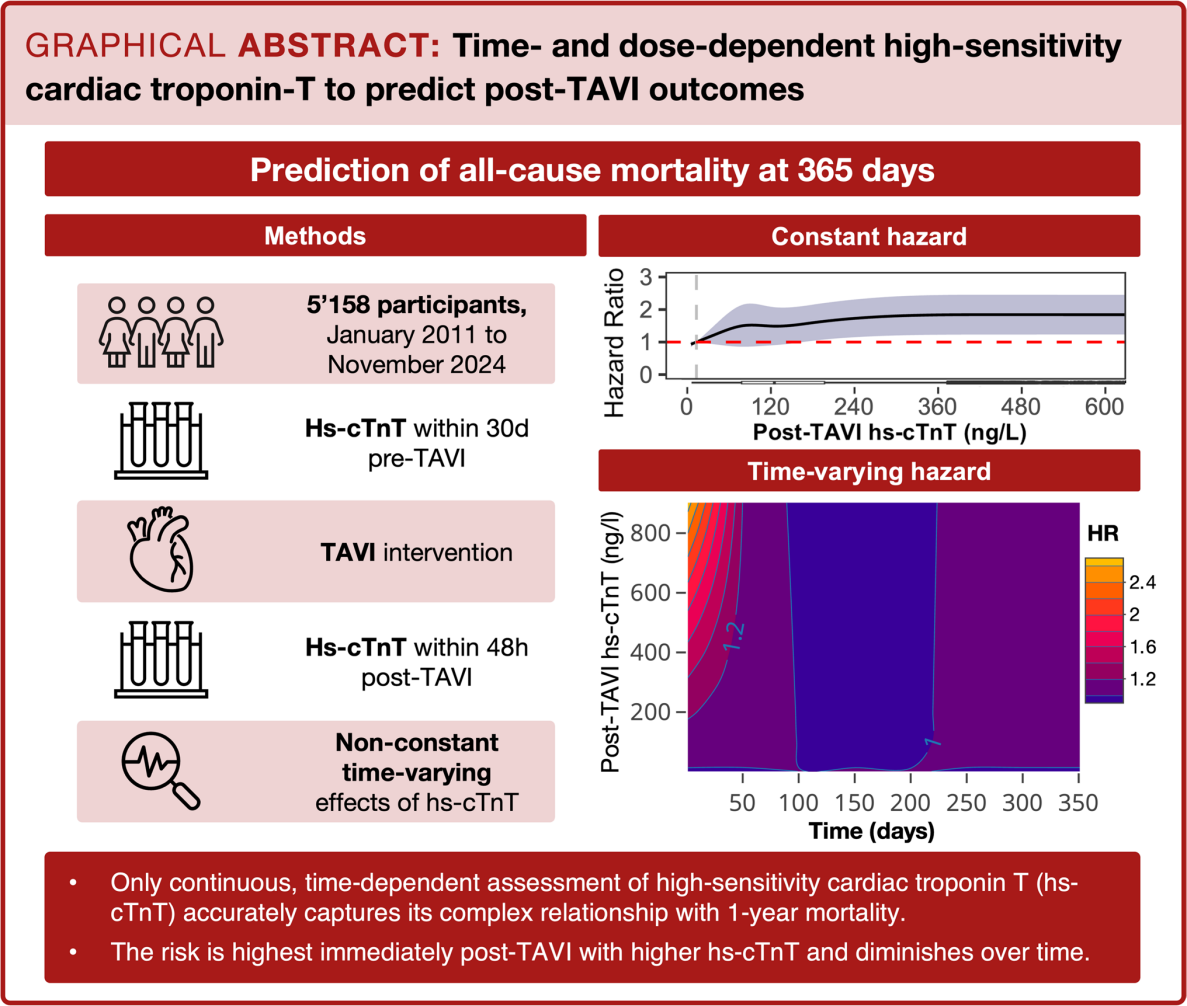

**Supplementary Information:**

The online version contains supplementary material available at 10.1007/s00392-025-02808-z.

## Introduction

Transcatheter aortic valve implantation (TAVI) is a safe and effective approach for the treatment of severe aortic stenosis, with recent data demonstrating favorable outcomes compared to surgical aortic valve replacement [1–3]. A possible complication of TAVI is periprocedural myocardial injury (PPMI), which may result from direct mechanical trauma to the myocardium, coronary microembolization, rapid pacing during valvuloplasty, or valve deployment causing transient coronary obstruction [4]. According to the Valve Academic Research Consortium (VARC) 3, PPMI is defined by an elevation in high-sensitivity cardiac troponin T (hs-cTnT) within 48 h post-procedure of ≥ 35 times the assay-specific upper reference limit (URL) of normal in patients with either new pathologic Q-waves in ≥ 2 contiguous leads or new left bundle branch block, or ≥ 70 times the URL in patients without ECG changes [5]. Several procedural and baseline patient characteristics including procedural access, device type, or renal function have been associated with PPMI. However, these associations have typically been modelled under the assumption of linear relationships [6–11]. PPMI has been shown to be a significant predictor of short- and long-term outcomes after TAVI [11–14]. Furthermore, pre- and post-TAVI hs-cTnT on their own have also been shown to be predictive of post-TAVI adverse events [15–20]. To date, most studies have employed a binary approach to PPMI using the VARC-3 criteria or have used arbitrary hs-cTnT cutoff values. This approach fails to capture time- and dose-dependent effects of hs-cTnT on outcomes, which limits the precision of risk stratification and subsequent patient management.

In this study, we aimed to identify non-linear predictors of post-TAVI hs-cTnT elevation, investigate the non-linear and time-dependent effects of pre- and post-procedural hs-cTnT concentrations on outcomes after TAVI, and compare these findings with the VARC-3 definition of PPMI.

## Methods

### Study design and patient cohort

Consecutive patients undergoing TAVI at two tertiary university centers (University Hospital Basel, Switzerland, and University-Heart Center Freiburg-Bad Krozingen, Germany) with available hs-cTnT measurements within 30 days pre- and 48 h post-procedurally were enrolled. All patients undergoing TAVI are included in prospective local databases that have been approved by local ethics committees and institutional review boards. Prior results from both registries have been reported elsewhere [21–23].

### Data collection, follow-up, and clinical endpoints

A web-based database was used to prospectively collect and store information on patients, including their baseline characteristics, procedural details, and follow-up data. Clinical follow-up information was acquired through structured interviews, documentation provided by referring physicians, and hospital discharge summaries. A designated clinical event committee systematically collected and evaluated all adverse events according to the VARC-3 criteria. The committee retrospectively adjudicated all reported endpoints based on the available documentation. PPMI was defined according to VARC-3 criteria as an increase of hs-cTnT within 48 h post-procedurally of ≥ 35 times the upper URL in patients with either new pathologic Q-waves in ≥ 2 contiguous leads or new left bundle branch block, or ≥ 70 times the URL in patients without ECG changes [5]. The primary endpoint was all-cause mortality at 1 year.

### Laboratory testing

As part of standard clinical blood testing, hs-cTnT was analyzed using heparin plasma specimens on the Elecsys 2010 hs-cTnT platform (Roche Diagnostics, Basel, Switzerland). This assay has a uniform 99th percentile of 14 ng/L with a 10% coefficient of variation at 13 ng/L. The limit of blank and limit of detection for this method have been established at 3 and 5 ng/L, respectively [24]. The current analysis incorporated all hs-cTnT concentrations obtained within 30 days before and 48 h after TAVI. When multiple measurements were available, the blood sample collected closest to the procedural time point was selected for analysis.

### Statistical analysis

Categorical variables are presented as count (percentage) and continuous variables as median (interquartile range (IQR)). Comparisons between continuous variables were performed using the Mann–Whitney *U* test. Categorical variables were compared using Pearson’s Chi-squared test or Fisher’s exact test, where appropriate. A multivariable linear regression model was fitted to identify predictors of post-TAVI hs-cTnT with selected variables being age, sex, body mass index (BMI), estimated glomerular filtration rate (eGFR), pre-TAVI hs-cTnT, procedural duration, main access site, valve size, and valve type. To allow for a non-linear effect, continuous variables were fitted with restricted cubic spline functions [19]. The assumptions of linear regression were checked with diagnostic plots. To satisfy them, post-TAVI hs-cTnT was subsequently log-transformed. Partial conditional effects plots were constructed to visualize the adjusted relationships between predictor variables and log-transformed hs-cTnT levels while holding other covariates constant. Three multivariable Cox proportional hazards models were fitted for hs-cTnT (pre- and post-TAVI) to investigate the potential association of hs-cTnT concentration and 1-year all-cause mortality. First, a model using dichotomized hs-cTnT according to VARC-3 criteria was used. Second, a model with continuous hs-cTnT but assuming linearity was fitted. Third, to avoid imposing linearity, we used a restricted cubic spline function to model the continuous non-linear association of hs-cTnT with the outcome [25]. Five spline knots were placed at 0.05, 0.275, 0.5, 0.725, and 0.95 percentiles of hs-cTnT marginal distribution, following Harrell’s recommendation [26]. Considering the total number of deaths and to avoid overfitting, seven independent variables were chosen for adjustment (age, sex, eGFR, presence of diabetes, hypertension, coronary artery disease, and New York Heart Association (NYHA) score), based on previous literature and matter-based knowledge. To assess whether associations differed by sex, between early and more recent procedures, or valve type, we conducted a sensitivity analysis including an interaction term between sex, procedure date, or valve type and hs-cTnT in the multivariable Cox proportional hazards models for all-cause mortality. We also repeated the analyses stratified by sex, procedure date, or valve type to examine if the relationship between hs-cTnT and outcomes varied between men and women, early and more recent procedures, and valve type. To relax the proportional hazards assumption, allowing for hazard ratios (HRs) to vary over time, multivariable Royston-Parmar flexible parametric survival models were fitted using the stpm2 function from the rstpm2 package [27]. In these models, hs-cTnT, relative changes therein, or *x*-fold increases from the URL (14 ng/L) were modelled with restricted cubic splines allowing for non-linear effects. Knots were placed at the 0.05, 0.275, 0.5, 0.725, and 0.95 percentiles of hs-cTnT marginal distribution. Time-dependent effects were incorporated by including interactions between hs-cTnT and the baseline log cumulative hazard function. HRs were calculated for each timepoint and hs-cTnT value relative to day 0 and a hs-cTnT of 14 ng/L or a relative change therein from pre- to post-procedurally of 1. Time-dependent HR plots, 3D mesh plots, and contour plots were constructed to visualize the non-proportional hazards [26]. All statistical analyses were performed using R 4.4.0 (R Foundation for Statistical Computing, Vienna, Austria). An interactive online tool was created to display specific HRs for user-specified post-TAVI hs-cTnT values and number of days post-TAVI. Used R packages are listed in the Supplemental material (Supplementary Table 1).

## Results

### Patient characteristics

Between January 2011 and November 2024, a total of 6178 patients were enrolled, out of which 5158 had both pre- and post-hs-cTnT measurements available. The median time of pre-TAVI hs-cTnT measurement was − 2.14 days (interquartile range (IQR) − 5.09, − 0.95) and the median time of post-TAVI hs-cTnT measurement was 1.48 h (IQR 0.82, 2.5). Patients had a median age of 82.7 years (IQR 79.0, 86.0), Euro SCORE II was 1.28% (IQR 0.79, 2.27), and STS calculated risk of mortality was 3.2% (IQR 2.1, 5.3). Pre- and post-TAVI hs-cTnT concentrations were 28 ng/L (IQR 17, 52) and 125 ng/L (IQR 78, 198), respectively (Table 1).

**Table 1:** Patient baseline characteristics

Variable	***N***	Overall, ***N*** = 5158^*I*^	Alive at 1 y,*** N*** = 4548^*I*^	Deceased at 1 y, ***N*** = 610^*I*^
Basic characteristics				
Sex	5158			
Male		2730 (53%)	2409 (53%)	321 (53%)
Female		2428 (47%)	2139 (47%)	289 (47%)
Age (years)	5158	82.7 (79.0, 86.0)	82.0 (79.0, 86.0)	83.0 (79.0, 87.0)
BMI (kg/m^2^)	5155	26.0 (23.5, 29.1)	26.1 (23.7, 29.1)	25.6 (22.7, 29.2)
Euro SCORE II (%)	4931	1.28 (0.79, 2.27)	1.23 (0.76, 2.19)	1.72 (0.97, 3.05)
STS calculated risk of mortality (%)	4602	3.2 (2.1, 5.3)	3.1 (2.0, 5.0)	4.4 (2.7, 7.0)
NYHA class	4947			
1		176 (3.6%)	160 (3.7%)	16 (2.7%)
2		1113 (22%)	1025 (23%)	88 (15%)
3		3120 (63%)	2758 (63%)	362 (62%)
4		538 (11%)	422 (9.7%)	116 (20%)
Preconditions				
Diabetes	5156	1487 (29%)	1277 (28%)	210 (34%)
Dyslipidemia	5158	2171 (42%)	1921 (42%)	250 (41%)
Hypertension	5158	2792 (54%)	2450 (54%)	342 (56%)
Peripheral artery disease	4950	1291 (26%)	1108 (25%)	183 (31%)
Coronary artery disease	4961	3062 (62%)	2684 (61%)	378 (65%)
Myocardial infarction	4970	715 (14%)	622 (14%)	93 (16%)
eGFR (Cockroft-Gault) (mL/min/1.73 m^2^)	5158	56 (41, 70)	57 (42, 70)	44 (31, 65)
Hemoglobin (g/dL)	5157	127 (115, 138)	128 (116, 139)	119 (107, 132)
Pre-TAVI hs-cTnT (ng/L)	5033	28 (17, 52)	27 (17, 48)	38 (23, 77)
Post-TAVI hs-cTnT (ng/L)	5158	125 (78, 198)	123 (77, 193)	141 (89, 233)
VARC-3 defined PPMI	5158			
PPMI		85 (1.6%)	65 (1.4%)	20 (3.3%)
Non-PPMI		5073 (98%)	4483 (99%)	590 (97%)
Previous interventions				
Any heart surgery	5158	370 (7.2%)	313 (6.9%)	57 (9.3%)
Prior pacemaker	5133	359 (7.0%)	290 (6.4%)	69 (11%)

*BMI*, body mass index; *NYHA*, New York Heart Association; *eGFR*, estimated glomerular filtration rate; *TAVI*, transcatheter aortic valve implantation; *hs-cTnT*, high-sensitivity cardiac troponin T; *VARC-3*, Valve Academic Research Consortium; PPMI, periprocedural myocardial injury

^*1*^*n* (%); median (IQR)

### Procedural characteristics

Most patients underwent TAVI via transfemoral access (97%) and received a self-expandable valve (63%). Median length of hospital stay was 7 days (IQR 5–10) (Table 2). There were no significant differences in procedural characteristics between men and women.

**Table 2:** Patient procedural characteristics

Variable	***N*** = 5158^*I*^
Procedural characteristics	
Access route	
Transfemoral	5004 (97%)
Non-transfemoral	154 (3.0%)
Valve size (mm)	26.0 (25.0, 29.0)
Valve type	
Mechanical-expandable	29 (3.9%)
Self-expandable	471 (63%)
Balloon-expandable	244 (33%)
Procedural outcomes	
In-hospital duration (days)	7.0 (5.0, 10.0)
Permanent pacemaker implantation	898 (20%)
Valve dislocation	21 (0.4%)
Cerebrovascular event	72 (1.4%)

^*1*^n (%); median (IQR)

### Predictors of post-procedural high-sensitivity cardiac troponin T elevations

**Fig. 1:**
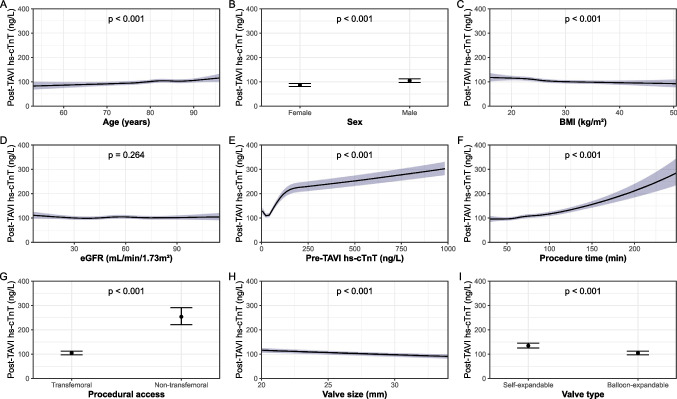
Partial conditional effect plots for predictors of log-transformed post-transcatheter-aortic valve implantation (TAVI) high-sensitivity cardiac troponin T (hs-cTnT). Continuous variables were modeled with restricted cubic splines. BMI, body mass index; eGFR, estimated glomerular filtration rate

Multiple linear regression analysis revealed significant positive associations for age, male sex, pre-TAVI hs-cTnT, procedural duration, non-transfemoral access, self-expandable valves, and elevated post-TAVI hs-cTnT (all *p* < 0.001). Higher BMI and valve size showed significant negative associations with elevated post-TAVI hs-cTnT (both *p* < 0.001) (Fig. 1).

### VARC-3 defined PPMI and continuous linear hs-cTnT values with proportional hazards

The median follow-up duration was 632 days (IQR 172, 1205). The rate of all-cause mortality after 1 year was 12% (Table 3). After an initial predominance of cardiovascular mortality, non-cardiovascular mortality increases in relevance, with both cardiovascular- and non-cardiovascular mortality having a similar, declining incidence (Supplementary Fig. 1). VARC-3 defined PPMI occurred in 80 out of 5158 patients (1.6%) and was not significantly associated with all-cause mortality (HR 1.56 (95% CI 0.99, 2.46, 2.46), *p* = 0.06). When hs-cTnT concentrations were analyzed as continuous linear variables — rather than dichotomized — and assuming proportional hazards, both elevated pre- and post-procedural hs-cTnT levels were independently associated with increased risk of 1-year all-cause mortality after pre-defined adjustment for age, sex, eGFR, presence of diabetes, hypertension, coronary artery disease, and NYHA score. Pre-procedural hs-cTnT was associated with an HR of 1.48 (95% CI 1.25, 1.75; *p* < 0.001), and post-procedural hs-cTnT with an HR of 1.34 (95% CI 1.16, 1.55; *p* = 0.001). The interaction of VARC-3 defined PPMI, pre- and post-procedural hs-cTnT concentration and sex, date of procedure, or valve type was non-significant in all cases.

**Table 3:** Incidence of specific outcomes at 1 year

Outcomes at 1 year	***N*** = 5158^*1*^
All-cause mortality	610 (12%)
Myocardial infarction	19 (0.4%)
Stroke	95 (1.8%)
Bleeding	226 (4.4%)
Reintervention	95 (1.8%)

^*I*^*n* (%)

### Continuous non-linear hs-cTnT and time-dependent hazard ratio analysis

**Fig. 2:**
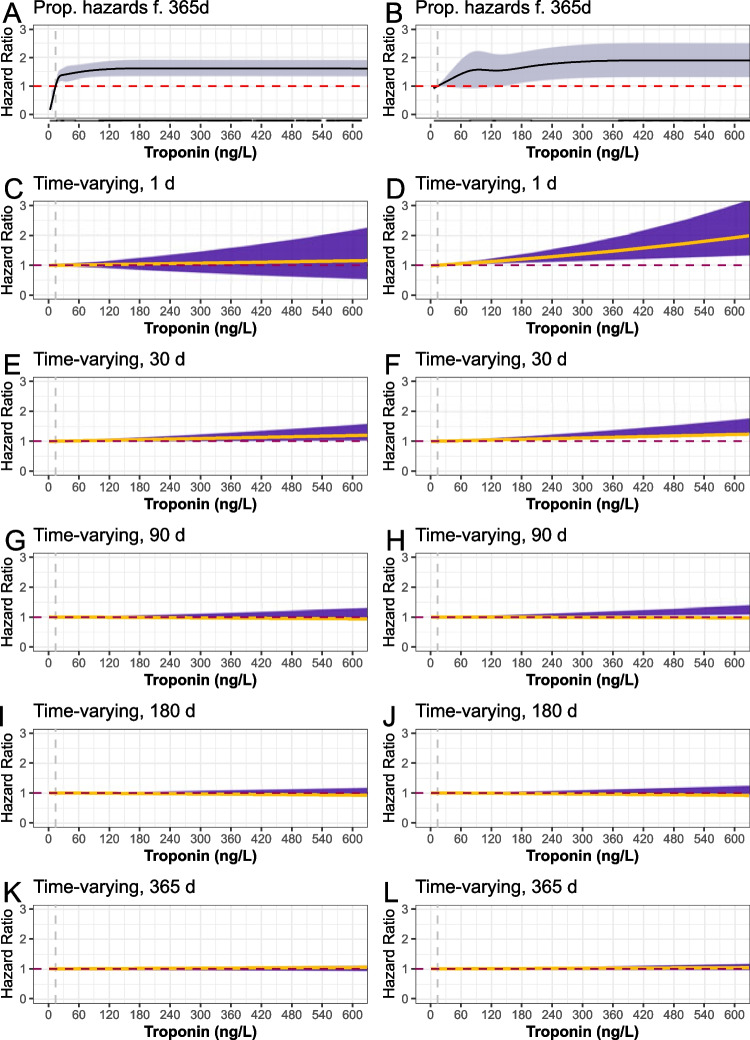
Hazard ratio (HR) plots when assuming proportional hazards during the whole 1-year follow-up (**A** and** B**) and relaxing the proportional hazard assumption (**C–L**), i.e., time-dependent HRs, for different follow-up timepoints for pre- (left column) and post-procedural (right columns) high-sensitivity cardiac troponin T (hs-cTnT). While the models assuming proportional hazards suggest a significant association between higher hs-cTnT and an elevated risk of all-cause mortality, time-dependent analyses reveal this effect to be limited to the immediate post-procedural time, with no significant differences shown to reference at later timepoints

**Fig. 3:**
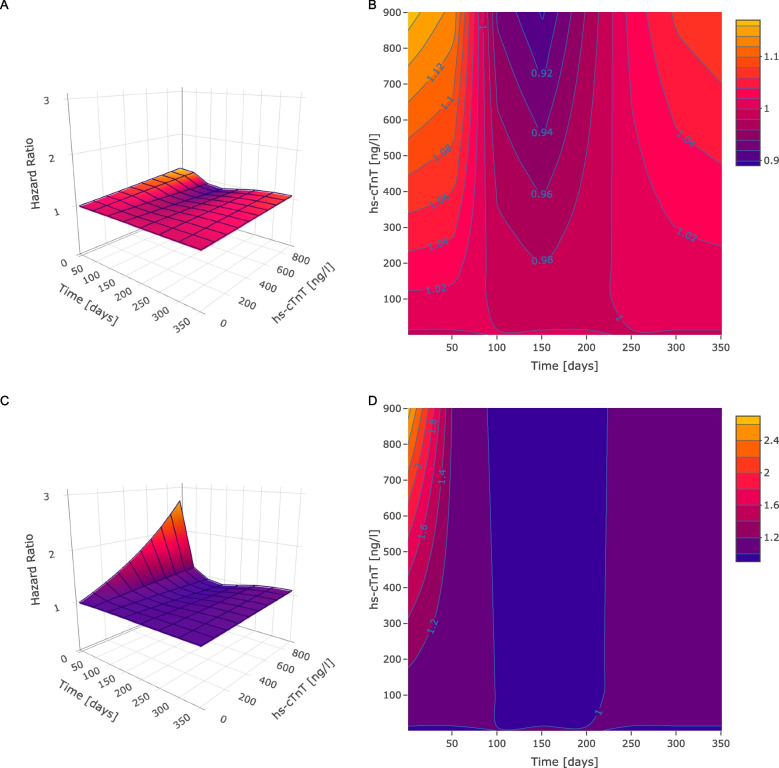
Time-dependent hazard ratios depicted as 3D mesh plots illustrating the predicted hazard ratio (HR) for all-cause mortality at 365 days and 2D contour plots for pre- (**A**, **B**) and post-transcatheter aortic valve implantation (TAVI) high-sensitivity cardiac troponin T (hs-cTnT) (**C**, **D**). The color gradient represents the HR magnitude. Pre-TAVI hs-cTnT shows a relatively constant hazard over time, while post-TAVI hs-cTnT demonstrates non-constant hazards, with the highest HR immediately post-I and higher post-TAVI hs-cTnT levels, that diminish over time

Modeling hs-cTnT as a non-linear continuous variable under the assumption of proportional hazards revealed a significant association between both pre- and post-TAVI hs-cTnT and all-cause mortality at 1 year (Fig. 2A and B). When relaxing the proportional hazards assumption to allow for time-dependent HRs, a more nuanced temporal risk pattern emerged. Pre-procedural hs-cTnT exhibited a relatively constant hazard over time. In contrast, post-procedural hs-cTnT, relative changes in hs-cTnT, and relative increases above the URL (14 ng/L) demonstrated non-constant hazards, where the hazard was highest early post-procedure for higher hs-cTnT, and diminished over time (Fig. 2C–L, Fig. 3; Supplementary Fig. 2, Supplementary Fig. 3). The duration of elevated risk was directly proportional to the magnitude of post-procedural hs-cTnT elevation. For example, patients with a post-procedural hs-cTnT of 600 ng/L remained at significantly increased risk for up to 2 months post-procedure, whereas patients with post-procedural hs-cTnT of 200 ng/L returned to baseline risk after approximately 20 days post-procedure, relative to a reference value of 14 ng/L. To facilitate clinical translation, an interactive online tool has been created that allows input of any post-TAVI hs-cTnT value and timepoint, generating both estimated HRs and graphical representations of the associated mortality risk (https://tstolte.shinyapps.io/TATroHRcalc/).

## Discussion

In this multicenter cohort study of 5158 patients investigating the time- and dose-dependent effects of hs-cTnT on outcomes after TAVI, we present several key findings: First, the VARC-3 criterion for PPMI following TAVI, which dichotomizes hs-cTnT, was not prognostic for all-cause mortality at 1 year. Second, modeling hs-cTnT as a linear or non-linear continuous variable under the assumption of proportional hazards demonstrated that both pre- and post-procedural hs-cTnT levels were associated with increased risk for all-cause mortality at 1 year. Third, time-dependent HRs for post-procedural hs-cTnT and relative changes therein predicted a more nuanced, non-constant hazard, with the highest hazard early post-procedure that declined over time.

The findings of this large multicenter study expand on prior evidence and carry potential clinical implications [6, 7, 10, 11, 15, 20, 28–32]. When dichotomized, post-TAVI hs-cTnT was not predictive of 1-year all-cause mortality. While several studies have demonstrated associations between VARC-3 criteria and post-TAVI outcomes [6, 11, 15, 20, 28–30], others have reported inconsistent findings [7, 10, 31, 32]. Several factors may explain these discrepancies. First, dichotomizing continuous variables reduces statistical power to identify an association by approximately one-third, [33] an issue exacerbated in studies with small effective sample sizes (e.g., low incidence of PPMI). Second, the prognostic relevance of specific hs-cTnT thresholds varies depending on the endpoint (all-cause mortality, cardiovascular death, or future MI) and timeframe (1 month vs. 1 year). Different informed thresholds will emerge depending on the statistical model and covariates included. Even a seemingly small distinction, such as using 60-day instead of 30-day all-cause mortality, can determine whether associations reach statistical significance. Notably, studies showing significant associations with dichotomized hs-cTnT had a mean follow-up of 650 days, compared to 281 days in studies showing no association. This may reflect the influence of accumulating non-procedural mortality, particularly in older cohorts (median of 82.7 years). Third, insufficient covariate adjustment can lead to an overestimation of the prognostic effect of hs-cTnT and the chosen endpoint [26].

To date, only Real et al. have analyzed hs-cTnT as a continuous variable, reporting post-TAVI hs-cTnT exceeding 81.8 times the URL predicted 1-year all-cause mortality when assuming proportional hazards [11]. Our findings corroborate these results, showing that relative increases exceeding 12-fold the URL were associated with elevated risk. Importantly, our analysis extends these findings by demonstrating nuanced time- and dose-dependent effects that reveal a more complex relationship between elevations in hs-cTnT and mortality risk.

The time-dependent nature of risk associated with post-procedural hs-cTnT represents a novel and clinically relevant finding. To our knowledge, no previous study has investigated the predictive value of hs-cTnT with a relaxed assumption of proportional hazards and as a continuous variable. The present findings demonstrate that the VARC-3 definition of PPMI, as well as hs-cTnT as a continuous variable, failed to reflect the complex time-dependent predictability of all-cause mortality. Importantly, the mortality risk associated with elevated post-procedural hs-cTnT was highest immediately after the procedure and gradually diminished over time, with the duration of elevated risk directly proportional to the magnitude of troponin elevation.

These findings challenge the current approach of using binary risk stratification based on the VARC-3 thresholds (≥ 35 × or ≥ 70 × URL). Our continuous modeling suggests that even minor hs-cTnT elevations, well below these thresholds, may carry prognostic information. This underlines the importance of minimizing myocardial injury during TAVI and argues against the reliance on arbitrary cutoffs. Several biological mechanisms may explain the time-dependent risk pattern. Acute myocardial injury during TAVI may trigger inflammatory cascades, leading to adverse remodeling, hemodynamic instability, or arrhythmogenic substrate formation [34, 35]. The substantial number of patients with elevated hs-cTnT at baseline may stem from advanced age in conjunction with high rates of cardiovascular comorbidities, such as coronary artery disease, arrhythmias, left ventricular hypertrophy, and chronic kidney disease. Higher post-TAVI hs-cTnT levels likely reflect more extensive myocardial damage, potentially caused by procedural complexity, such as reduced ejection fraction, prolonged ventricular rapid pacing, or transapical access. The gradual decline in risk over time may represent a weakening of the prognostic ability of post-TAVI hs-cTnT and myocardial injury, possibly driven by causes of death becoming more heterogeneous with the passing of time, or physiological myocardial recovery processes and adaptation to the initial injury [36]. Consistent with this, early deaths were predominantly cardiovascular and the prognostic separation by post‑TAVI hs‑cTnT attenuated thereafter. Alternatively, the highest-risk patients with substantial PPMI may experience early mortality, leading to survivor bias in later follow-up periods. Further mechanistic studies are needed to explore the underlying causes of the adverse effects of myocardial injury.

The absence of a statistically significant interaction in hs-cTnT levels and sex for all-cause mortality corroborates previous literature on hs-cTnT levels and PPMI in TAVI patients [6, 9, 29]. It does not imply that there is no sex-based difference in mortality risk but rather indicates that the association between hs-cTnT levels and mortality is consistent across sexes. The time-dependent nature of risk associated with post-procedural hs-cTnT is a novel finding with substantial clinical implications. Patients with higher hs-cTnT elevations not only faced increased mortality risk but remained at elevated risk for longer than comparable patients with lower hs-cTnT: up to 2 months with very high hs-cTnT values (600 ng/mL) compared to only 20 days with moderate elevations (200 ng/mL). This temporal pattern suggests that post-TAVI surveillance strategies should be tailored to the degree of myocardial injury. Patients at higher risk could benefit from more frequent, potentially imaging-guided monitoring to assess the recovery of the damaged myocardium and to enable early detection of health deterioration. To facilitate clinical implementation of these findings for patient-tailored decision-making, our interactive online tool allows the input of specific post-TAVI hs-cTnT values and time since TAVI to obtain individualized HRs and visual representations for the immediate risk predicted at this timepoint by post-TAVI hs-cTnT (https://tstolte.shinyapps.io/TATroHRcalc/).

Some limitations of our study should be acknowledged. The present study exclusively included patients with available high-sensitivity cardiac troponin T, and did not evaluate other assays for troponin I. This limits the generalizability of our findings to alternative troponin assays. Additionally, we did not explore all potential mechanisms linking PPMI to mortality, and some unmeasured confounders may remain despite our covariate adjustments. Furthermore, we did not validate our findings in a separate external cohort. Nevertheless, the results from our study are derived from the by far largest multicenter dataset investigating the prognostic relevance of hs-cTnT elevations in patients undergoing TAVI. Future studies should investigate whether targeted interventions and more intensive follow-up care in patients with elevated hs-cTnT levels can improve outcomes.

## Conclusions

The VARC-3 binary definition of PPMI was not prognostic for all-cause mortality and failed to reflect the time-dependent risk associated with myocardial injury after TAVI. Higher levels of post-procedural hs-cTnT were associated with a prolonged period of increased mortality risk, with the duration ranging from days to months when using the URL of hs-cTnT (14 ng/L) as a reference. These findings support a paradigm shift from the binary to dynamic, individualized risk assessment incorporating time-dependent and non-linear effects of hs-cTnT. This refined understanding has the potential to enhance clinical decision-making by tailoring post-procedural surveillance to individual patient risk profiles.

## Clinical perspective

### What is known?

Transcatheter aortic valve implantation (TAVI) is an increasingly common and effective treatment for severe aortic stenosis, but periprocedural myocardial injury (PPMI), detectable through elevated high-sensitivity cardiac troponin T (hs-cTnT), remains a concern. Currently, PPMI diagnosis relies on fixed cutoffs defined by the Valve-Academic-Research Consortium-3, which do not fully reflect the complex relationship between hs-cTnT levels, time post-procedure, and patient outcomes. These fixed thresholds limit precision in risk stratification and personalized management following TAVI.

### What is new?

This large multicenter study of 5158 patients demonstrates that the VARC-3 definition of PPMI lacks prognostic value for 1-year all-cause mortality after TAVI. Modeling hs-cTnT as a continuous variable and accounting for time-dependent effects, however, revealed a dynamic risk trajectory: higher hs-cTnT levels correlated with increased mortality risk, particularly in the early post-procedural period, with this risk diminishing over time. An interactive online tool was created to facilitate clinical translation of these findings.

### What is next?

Future research should focus on validating these findings in external cohorts and exploring whether targeted interventions based on continuous, time-dependent hs-cTnT levels can improve patient outcomes after TAVI. Integrating dynamic hs-cTnT into existing risk prediction models could enhance clinical decision-making and personalize post-procedural surveillance strategies to individual patient risk profiles, leading to improved care.

## Supplementary Information

Below is the link to the electronic supplementary material.

## Supplementary Information

Below is the link to the electronic supplementary material.

Supplementary File 1 (14.1 MB)

## Data Availability

Data that support the findings of this study are available upon reasonable request from the corresponding author.

## Abbreviations

BMI: Body mass index
CI: Confidence interval
ECG: Electrocardiogram
eGFR: Estimated glomerular filtration rate
HRs: Hazard ratios
hs-cTnT: High-sensitivity cardiac troponin T
IQR: Interquartile range
NYHA: New York heart association
PPMI: Periprocedural myocardial injury
SD: Standard deviation
TAVI: Transcatheter aortic valve implantation
URL: Upper reference limit
VARC: Valve academic research consortium

## Declarations

### Disclaimer

The manuscript and its contents have not been published previously and are not being considered for publication elsewhere in whole or in part in any language, including publicly accessible websites or e-print servers.
