# Supplementary material for: Time- and dose-dependent high-sensitivity cardiac troponin-T to improve outcome prediction after TAVI: a multicenter cohort study
Source: Clin Res Cardiol. 2025 Nov 24;115(7):1241–52. doi: 10.1007/s00392-025-02808-z (PMC13249652; doi:10.1007/s00392-025-02808-z)

**Time- and dose-dependent high-sensitivity cardiac troponin-T to predict outcomes after TAVI: a multi-center cohort study**

**SUPPLEMENTARY**

**SUPPLEMENTARY TABLES**

**Supplementary Table 1:** Packages used in statistical analysis

| **Package name** |
| --- |
| cowplot |
| dplyr |
| forcats |
| ggplot2 |
| glue |
| gt |
| gtsummary |
| haven |
| Hmisc |
| lubridate |
| patchwork |
| plotly |
| purrr |
| RColorBrewer |
| readr |
| readxl |
| rms |
| rstpm2 |
| stringr |
| survival |
| survPen |
| tibble |
| tidyr |
| tidyverse |

**SUPPLEMENTARY FIGURE LEGEND**

**Supplementary Figure 1:** Incidence of cardiovascular and non-cardiovascular mortality within 1 year after transcatheter aortic valve implantation (TAVI)

**Supplementary figure 2**: Hazard ratio (HR) plots when assuming proportional hazards (A and B) and relaxing the proportional hazard assumption (C – L), i.e. time-varying HRs, for relative changes in high-sensitivity cardiac troponin T (hs-cTnT) from pre-to post transcatheter aortic valve implantation (TAVI) (**left column**) & relative levels of the upper reference level (URL, 14 ng/L) of post-TAVI hs-cTnT (**right column**)


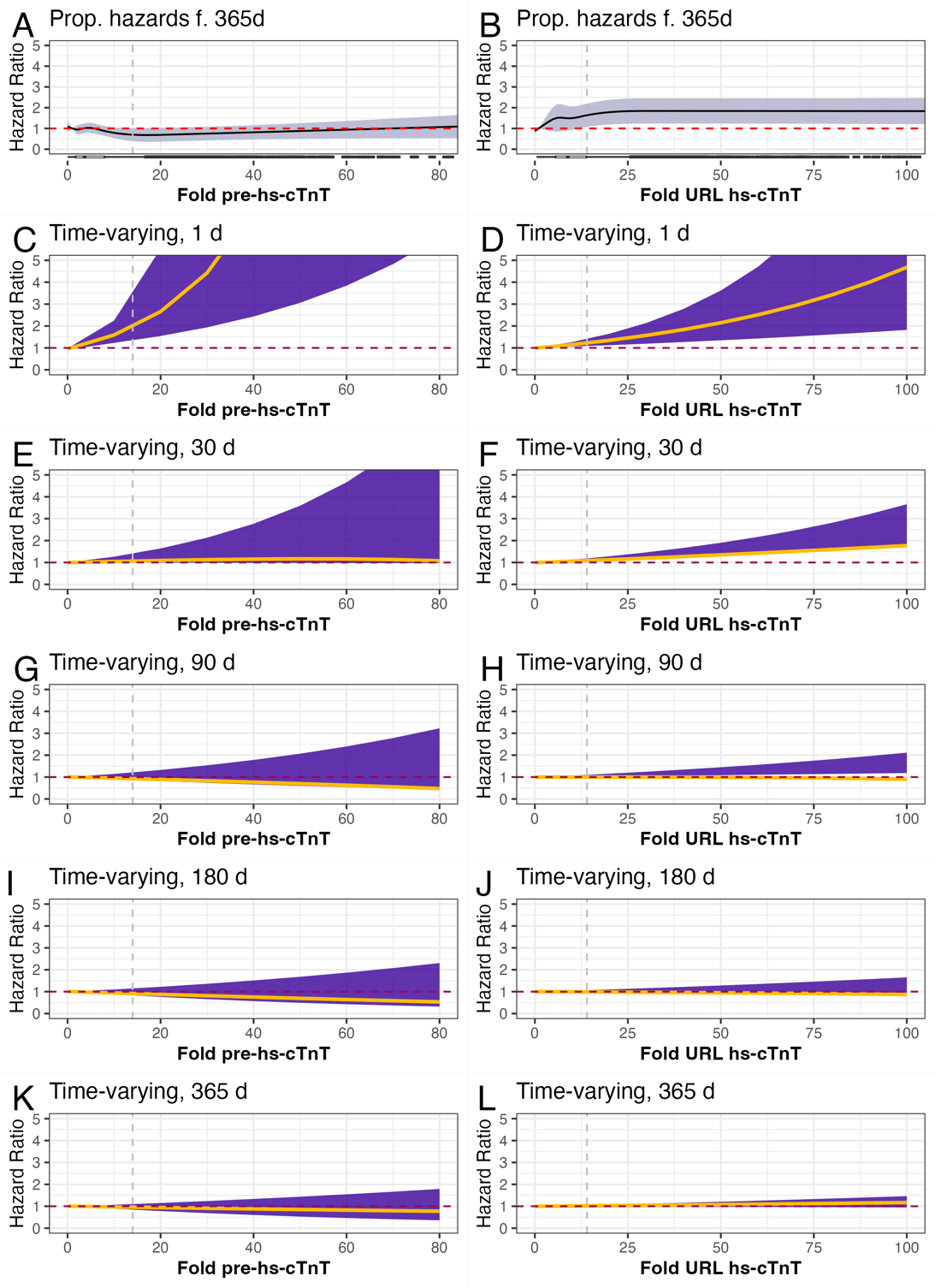


**Supplementary Figure 3:** Time-varying hazard ratios depicted as 3D mesh plots illustrating the predicted hazard ratio (HR) for all-cause mortality at 365 days and 2D contour plots for changes in high-sensitivity cardiac troponin T (hs-cTnT) from pre-to post transcatheter aortic valve implantation (TAVI) (**A, B**) & relative levels of the upper reference level (URL, 14 ng/L) of post-TAVI hs-cTnT (**C, D**). The color gradient represents the HR magnitude. Both demonstrate non-constant hazards, with the highest HR immediately post-TAVI and higher post-TAVI hs-cTnT, that diminish over time.
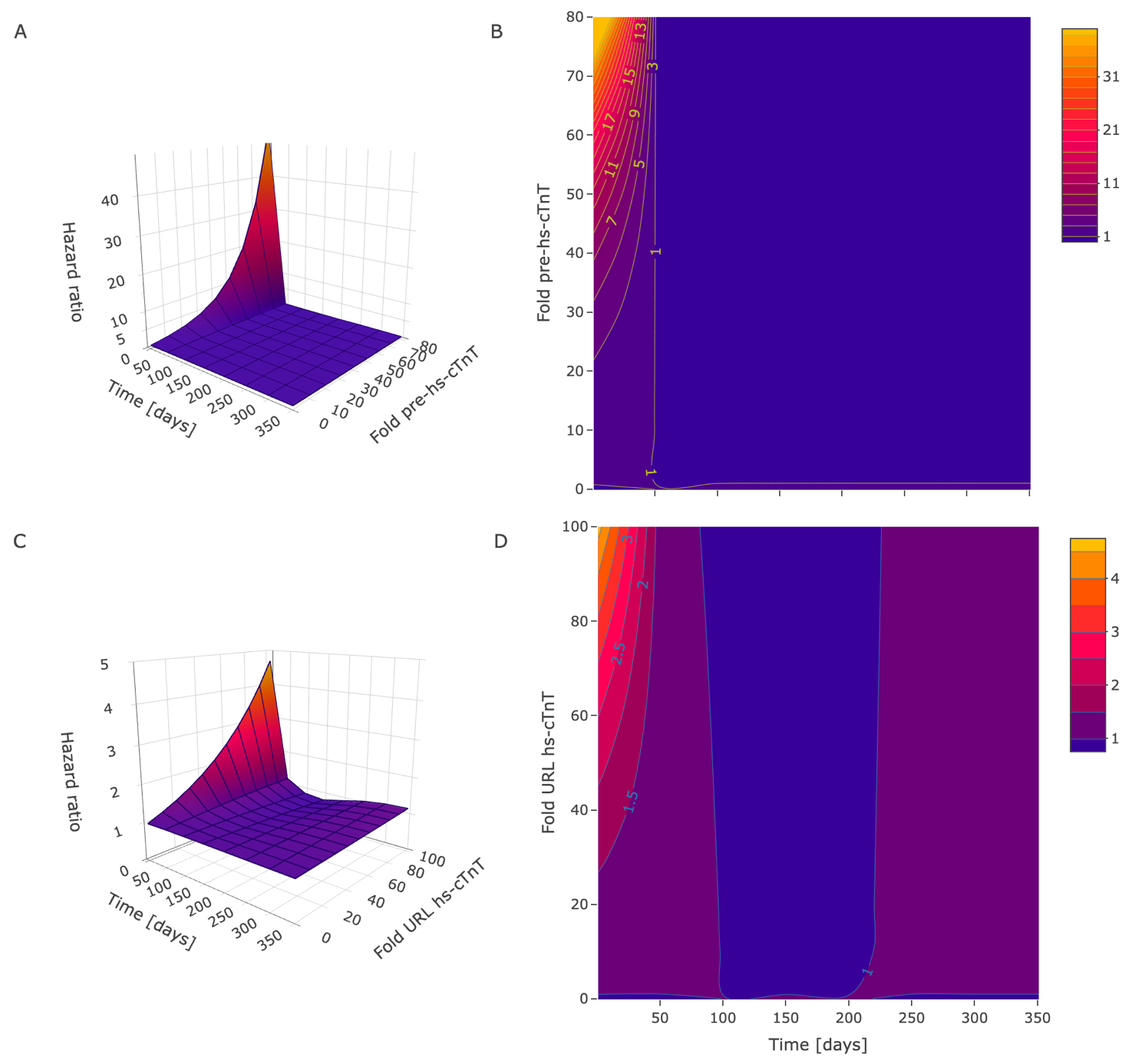

Supplement: Supplementary file 1 — (14.1 MB) [file 392_2025_2808_MOESM1_ESM.docx]
